# Increased Expression of Flotillin-2 Protein as a Novel Biomarker for Lymph Node Metastasis in Nasopharyngeal Carcinoma

**DOI:** 10.1371/journal.pone.0101676

**Published:** 2014-07-11

**Authors:** Qiuyuan Wen, Jiao Li, Weiyuan Wang, Guiyuan Xie, Lina Xu, Jiadi Luo, Shuzhou Chu, Lei She, Duo Li, Donghai Huang, Songqing Fan

**Affiliations:** 1 Department of Pathology, the Second Xiangya Hospital, Central South University, Changsha, Hunan, China; 2 Department of Oncology, the Second Xiangya Hospital, Central South University, Changsha, Hunan, China; 3 Department of Otorhinolaryngology, Xiangya Hospital, Central South University, Changsha, Hunan, China; The University of North Carolina at Chapel Hill, United States of America

## Abstract

Nasopharyngeal carcinoma (NPC) is a head and neck malignant tumor rare throughout most of the world but common in Southeast Asia, especially in Southern China. Flotillin-2 (Flot-2) is not only an important component of cellular membrane, but also involves in various cellular processes such as membrane trafficking, T cell and B cell activation, regulation of several signaling pathways associated with cell growth and malignant transformation, keeping structure and junction of epidermal cells and formation of filopodia. Although such molecular effects of Flot-2 have been reported, whether the expression of Flot-2 protein is associated with clinicopathologic implication for NPC has not been reported. The purpose of this research is to investigate the expression of Flot-2 protein in NPC and control nasopharyngeal epithelial tissues by immunohistochemistry and elucidate the association between the expression of Flot-2 protein and clinicopathological characteristics of NPC. The results showed that the positive percentage of Flot-2 expression in the NPC, nasopharyngeal epithelia with atypical hyperplasia and in the control nasopharyngeal mucosa epithelia was 88.8% (119/134), 76.9% (10/13) and 5.7% (5/88), respectively. There was significantly higher expression of Flot-2 protein in NPC and nasopharyngeal epithelia with atypical hyperplasia compared to the control nasopharyngeal mucosa epithelia (*P*<0.001, respectively). The positive percentage of Flot-2 protein expression in NPC patients with lymph node metastasis was significantly higher than those without lymph node metastasis. Increasing of Flot-2 expression was obviously correlated with clinical stages of NPC patients. The expression of Flot-2 was proved to be the independent predicted factor for lymph node metastasis by multivariate analysis. The sensitivity of Flot-2 for predicting lymph node metastasis of NPC patients was 93%. Taken together, our results suggest that the increased expression of Flot-2 protein is a novel higher sensitivity biomarker that can predict lymph node metastases in NPC.

## Introduction

Nasopharyngeal carcinoma (NPC) is a head and neck malignant tumor rare throughout most of the world but common in Southeast Asia, especially in Southern China, which is derived from nasopharyngeal epithelium with characteristics of early cervical lymph node metastasis and high incidence rate of distant metastasis. Distant metastasis and local recurrence still are main causes which result in the failure to cure NPC. Epstein-Barr virus (EBV) infection, chemical carcinogens, genetic variation or spontaneous mutation are closely associated with its occurrence and development [1]–[3]. Identification of the genes that are crucial for metastatic dissemination is of great value in NPC. Therefore, further elucidation of the molecular mechanism underlying NPC metastasis is essential for the development of new effective therapeutic agents.

Flotillin-2 (Flot-2) is a highly conserved protein and belongs to the SPFH (Stomatin/Prohibitin/Flotillin/HflK/C) protein superfamily, which is initially identified as a protein that is up-regulated during axon regeneration after optic nerve lesion [4]–[5]. It is a major protein from caveolae/lipid raft and is involved in epidermal cell adhesion and also plays an important role in inducing the formation of filopodia [5]. Over-expression of Flot-2 has been reported to be associated with human melanoma progression and lymph node metastasis [6]–[7]. Recently, it is reported that over-expression of Flot-2 is associated with poor prognosis and reduced survival of breast cancer patients with both early-and late-stage [8]. Also, Flot-2 is an independent prognostic factor and a potential novel biomarker for lymph node metastasis in gastric cancer [9]. It is reported that there is a higher expression of Flot-2 in metastatic NPC cells than in non-metastatic NPC cells [10]. However, whether the alteration of the expression of Flot-2 protein is associated with development and progression or clinicopathologic/prognostic implication for NPC has not been reported. In the present study, we have investigated the expression of Flot-2 protein in 134 cases of NPC, 13 cases of nasopharyngeal epithelial specimens with atypical hyperplasia and 88 cases of control nasopharyngeal epithelial specimens by Immunohistochemistry (IHC). We found that the positive percentage of Flot-2 protein expression in NPC and the nasopharyngeal epithelia with atypical hyperplasia was significantly higher than that in the control nasopharyngeal mucosa epithelia. Also, higher expression of Flot-2 is associated with the lymph node metastasis and clinical stages in NPC. Therefore, our results indicate that Flot-2 plays important roles in the progression and metastasis of NPC. Moreover, multivariate analysis confirmed that increased expression of Flot-2 protein might be an independent predictive factor for lymph node metastasis in NPC.

## Materials and Methods

### Ethics Statement

Samples were obtained with informed consent and all protocols were approved by the Second Xiangya Hospital of Central South University Ethics Review Board (Scientific and Research Ethics Committee, no. S06/2000). Written informed consent was obtained from all patients. Also the written informed consent was obtained from the next of kin, caretaker, or guardians on the behalf of the minors/children participants involved in our study.

### Tissue samples and clinical data

One hundred and thirty-four (134) paraffin-embedded NPC cases from the primary NPC patients with their age from 17 to 76 year (median, 47.24 years), also 13 cases of nasopharyngeal epithelia with atypical hyperplasia and 88 cases of control nasopharyngeal epithelial specimens from independent patients with chronic inflammation of nasopharyngeal mucosa, were obtained from the Second Xiangya Hospital of Central South University (Changsha, China). No patient had previously been treated with chemotherapy and radiotherapy at the time of original biopsy. Complete clinical record and follow-up data were available for all patients. Written informed consent was obtained from all patients, and this study was approved by the Ethics Review Committee of the Second Xiangya Hospital of Central South University. All specimens had been confirmed pathological diagnosis according to the 2005 WHO histological classification of NPC. The patients were staged according to the UICC/AJCC 1997 staging system of NPC.

The histological patterns and clinical stages of NPC were classified as follows: 121 cases of undifferentiated carcinomas and 13 cases of differentiated non-keratinizing carcinomas; 6 cases of clinical stage I, 42 cases of stage II, 50 cases of stage III, and 36 cases of stage IV. Among these patients included in the research, 100 patients were positive for cervical lymph node metastasis (N1/N2/N3 = 100) and 34 patients were negative (N0 = 34). Among 134 patients, 5 patients were treated by chemotherapy alone, 62 patients by radiotherapy alone, and 66 patients by combined radiotherapy and chemotherapy. Complete clinical record and follow-up data of all patients were available. Overall survival time was calculated from the data of diagnosis to the date of death or the data last known alive. A total of 82 patients (61.2%) were alive with a mean follow-up period of 59.2 months (3–120months).

### IHC and Scores

The IHC staining for Flot-2 protein in NPC sections was carried out using ready-to use Envision TM ^+^ Dual Link System-HRP methods(Dako; Carpintrria, CA). The staining condition for antibody was adjusted according to our laboratory experience. Briefly, each NPC section was deparaffinized and rehydrated, and high-temperature antigen retrieval was achieved by heating the samples in 0.01 M citrate buffer in a domestic microwave oven at full power(750 Watts) for 30 minutes, then the samples were immersed into methanol containing 0.3%H_2_O_2_ to inactivate endogenous peroxidase at 37°C for 30 minutes. To eliminate nonspecific staining, the slides were incubated with appropriate preimmune serum for 30 minutes at room temperature. After incubation with a 1∶1000 dilution of primary antibody to Flot-2 protein (Rabbit polyclonal antibody, Catalog: #S0051, Epitomics, Inc.) at 4°C overnight, slides were rinsed with phosphate-buffered saline (PBS) and incubated with a labeled polymer-HRP was added according to the manufacturer's instructions and incubated 30 minutes. Color reaction was developed by using 3, 3′-diaminobenzidine tetrachloride (DAB) chromogen solution. All slides were counterstained with hematoxylin. Positive control slides were included in every experiment in addition to the internal positive controls. The specificity of the antibody was determined with matched IgG isotype antibody as a negative control.

Two pathologists who were blinded to the IHC scores separately carried out the data analysis as described previously [11]. The Flot-2 staining were scored as negative (<10% staining) and positive (≥10% staining).

### Statistical analyses

All statistical analyses were performed using SPSS 19.0. The chi-square test was used to analyze the relationship between the expression of Flot-2 protein and clinicopathological characteristics of NPC. Kaplan-Meier analysis was performed for overall survival curves and statistical significance was assessed using the log-rank test. Overall survival was defined as the time from the treatment initiation (diagnosis) to the date of death. To identify whether expression of Flot-2 protein is an independent factor for lymph node metastasis of NPC, the multivariate logistic regression analysis was performed. The sensitivity, specificity, positive predictive value, negative predictive value and agreement rate of Flot-2 staining were hired to evaluate the validation of Flot-2 in predicting lymph node metastasis of NPC patients. All *P* values were based on the two-sided statistical analysis and p-value less than 0.05 was considered to be statistically significant.

## Results

### Association between expression of Flot-2 protein and clinicopathological features of NPC

**Figure 1:**
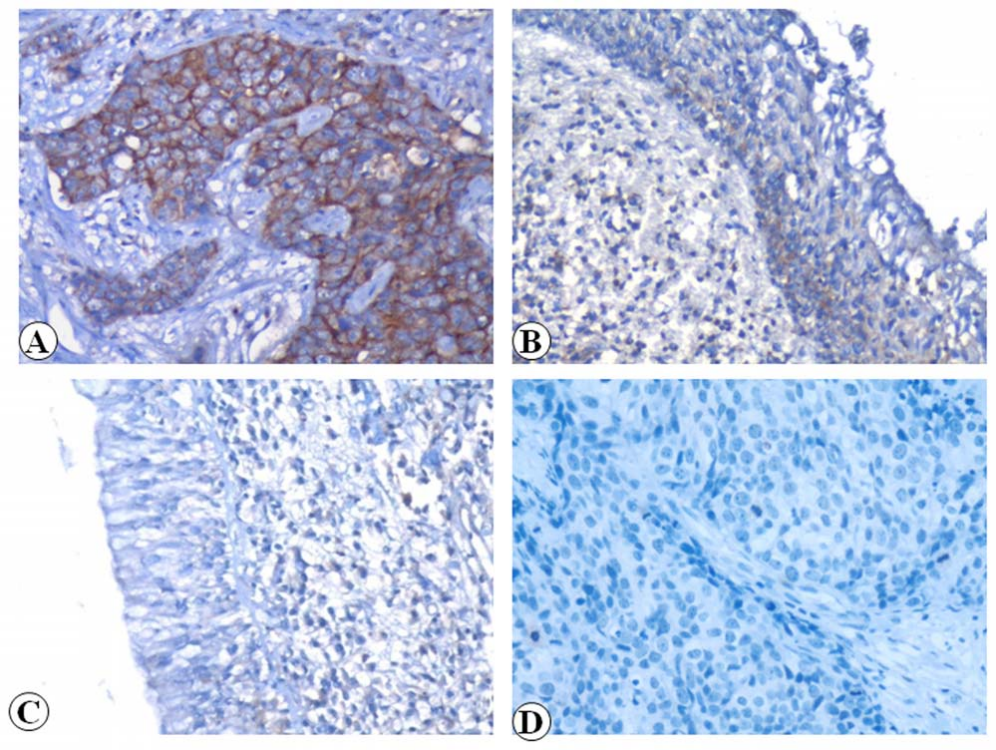
Expression of Flot-2 protein in NPC, atypical hyperplasia epithelial cells and control nasopharyngeal epithelial cells was detected by IHC. The expression of Flot-2 protein was detected by immunohistochemistry using specific antibody as described in the section of materials and methods. Strong positive expression of Flot-2 protein was found in NPC cell membranes (Fig 1A, 20×, IHC, DAB staining). Weak Flot-2 membrane-staining was showed in the atypical hyperplasia epithelial cells (Fig 1B, 20×, IHC, DAB staining). Negative staining of Flot-2 was showed that in the control nasopharyngeal epithelial cells (Fig 1C, 20×, IHC, DAB staining). Negative control showed no Flot-2 staining in the NPC cells (Fig 1D, 20×, IHC, DAB staining).

**Figure 2:**
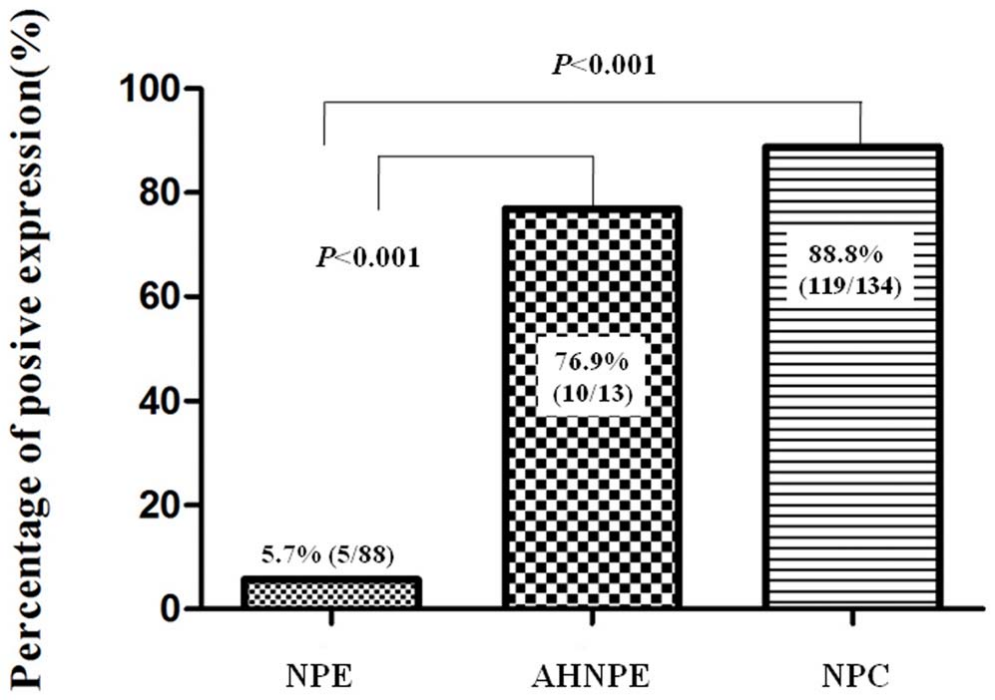
Expression of Flot-2 protein in NPC and atypical hyperplasia nasopharyngeal epithelia compared to the control nasopharyngeal mucosa epithelia. Results showed that there were significant differences between the groups which were statistically evaluated by chi-square test.

We examined the positive expression and cellular location of Flot-2 in NPC, atypical hyperplasia nasopharyngeal epithelial specimens and control of normal nasopharyngeal epithelial tissues by IHC. High positive expression of Flot-2 protein (Fig 1A) was identified on the NPC cell membranes and weak Flot-2 membrane-staining was found in the atypical hyperplasia nasopharyngeal epithelial cells (Fig 1B). Also, Fig 1C showed that there was no Flot-2 staining in the nasopharyngeal epithelial cells. Negative control showed no Flot-2 staining in the NPC cells (Fig 1D,). The positive percentage of Flot-2 expression in NPC, nasopharyngeal epithelia with atypical hyperplasia and control nasopharyngeal epithelial tissues was 88.8% (119/134), 76.9% (10/13) and 5.7% (5/88), respectively. There was significantly higher expression of Flot-2 protein in NPC and nasopharyngeal epithelia with atypical hyperplasia compared to the control nasopharyngeal mucosa epithelia (*P*<0.001, respectively) (Fig 2).

We further analyzed the associations between expression of Flot-2 protein and NPC clinicopathological features including patients' age, gender, clinical stages, histological type, lymph node metastasis, and survival status by univariate Chi-Square Test. Date shown in Table 1 indicated that NPC with lymph node metastasis presented higher positive expression of Flot-2 protein than those without metastasis (*P* = 0.008). The results also indicated a strong positive correlation between positive expression of Flot-2 and clinical stages of NPC, the advanced clinical stages (stage III and IV) had significantly higher positive percentage of Flot-2 expression than that in the early stages of NPC (stage I and II) (P = 0.038). However, no correlation was found between the positive expression of Flot-2 protein and age, gender, histological type and survival status of NPC patients (P>0.05, respectively).

**Table 1:** Analysis of the association between expression of Flot-2 protein and clinicopathological features of NPC.

	Flot-2 expression		
Clinicopathological features (n)	Positive (%)	Negative (%)	*P*-value
**Age**			
≤40 years(n = 50)	41(82.0)	9(18)	
>40 years(n = 84)	78(92.9)	6(7.1)	0.054
**Gender**			
Male(n = 104)	91(87.5)	13(12.5)	
Female(n = 30)	28(93.3)	2(6.7)	0.372
**Clinical stages**			
**Stage**_I-II_ (n = 48)	39(81.3)	9(18.8)	
**Stage**_III-IV_ (n = 86)	80(93.0)	6(7.0)	0.038*
**Lymph node status**			
LNM (n = 100)	93(93.0)	7(7.0)	
No LNM (n = 34)	26(76.5)	8(23.5)	0.008*
**Histological type**			
UDC (n = 121)	108(89.3)	13(10.7)	
DNKC (n = 13)	11(84.6)	2(15.4)	0.614
**Survival status**			
Alive (n = 82)	72(87.8)	10(12.2)	
Death (n = 52)	47(90.4)	5(9.6)	0.644

*Chi-square test, statistically significant difference (*P*<0.05).

UDC: Undifferentiated carcinoma; DNKC: Differentiated non-keratinized carcinoma. LNM: lymph node metastasis.

### Impact of expression of Flot-2 protein on the prognosis of NPC patients

To further examine the impact of expression of Flot-2 protein on the survival status of NPC patients, we employed the Kaplan-Meier analysis to plot the survival curve of all 134 NPC patients, and statistical significance was assessed using the log-rank test. At the time of analysis, the number of NPC patient specific deaths was 52(38.8%).

**Figure 3:**
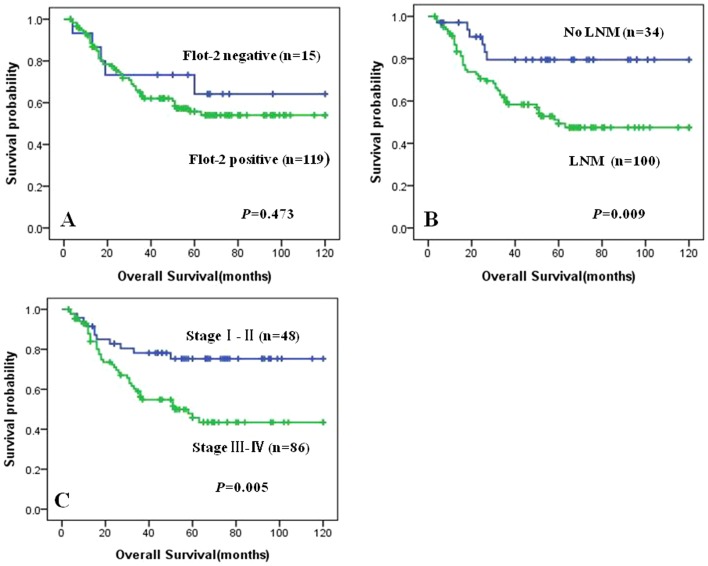
Kaplan-Meier curve for overall survival of NPC patients with expression of Flot-2 and different clinicopathological characteristics. Kaplan-Meier analysis to plot the overall survival curves of all 134 NPC patients with expression of Flot-2 and different clinicopathological characteristics and statistical significance was assessed using the long-rank test. Fig 3A Expression of Flot-2 protein in the NPC patients was no significantly related to their prognosis (P>0.05, two sided). Fig 3B NPC patients with lymph node metastasis were significantly related to poor prognosis compared to those patients without lymph node metastasis (P = 0.009, two sided). Fig 3C NPC patients with clinical stage III-IV were significantly related to poor prognosis compared to those patients with clinical stage I-II (P = 0.005, two sided).

Figure 3 illustrated the Kaplan-Meier survival plots for NPC patients with different expression of Flot-2 protein (Figure 3A). Univariate survival (long-rank test) analysis showed that there was no significant difference in the overall survival rates between NPC patients with positive expression of Flot-2 protein and these Flot-2-negative NPC patients (*P* = 0.473).

We also plotted the survival curves for NPC patients with the conventional prognostic parameters, including clinical stages, lymph nodal status. As shown in Figure 3 B–C, absence of lymph node metastasis or lower clinical stages (stage I and II) had a positive impact on the overall survival rates of NPC. The NPC patients with lymph node metastasis and advanced stage NPC (stage III and IV) had lower overall survival than that patients without lymph node metastasis and with early stage NPC (stage I and II) (P = 0.009, Figure 3B, and P = 0.005, Figure 3C, respectively).

To determine whether expression of Flot-2 protein was the independent predicted parameter for lymph node metastasis in NPC, a multivariate logistic regression analysis was carried out to further evaluate the expression of Flot-2 as the lymph node metastasis factor. As mentioned in Table 2, the positive expression of Flot-2 protein and clinical stages were significantly correlated with lymph node metastasis of NPC patients (*P* = 0.031, *P* = 0.023, respectively, Table 2). Again, no effect was detected with age, gender and histological type of NPC (*P*>0.05 for all). These results of multivariate analysis proved that high expression of Flot-2 protein was an independent factor for lymph node metastasis of NPC regardless of clinical stages, histological type, age and gender.

**Table 2:** Multivariate logistic regression analysis of lymph node metastasis factors in NPC patients.

Variables	B	S.E.	Wald	Sig.	Exp(B)	95.0%CI for Exp(B)	
						Lower	Upper
Age(years)	0.152	0.438	0.120	0.729	1.164	0.493	2.746
Gender	−0.830	0.479	3.004	0.083	0.436	0.171	1.115
Clinical stage	0.974	0.427	5.197	0.023*	2.648	1.146	6.116
Histological patterns	0.332	0.750	0.196	0.658	1.394	0.321	6.061
Flot-2 expression	1.314	0.607	4.676	0.031*	3.719	1.131	12.233

95% CI: 95%confidence interval.

*P<0.05.

### Validation of Flot-2 in predicting lymph node metastasis of NPC patients

In order to evaluate the validation of Flot-2 in predicting lymph node metastasis of NPC patients, the sensitivity ( =  true positive/[true positive + false negative]), specificity ( =  true negative/[false positive + true negative]), positive predictive value ( =  true positive/[true positive + false positive]), and negative predictive value ( =  true negative/[true negative + false negative]), agreement rate ( =  [true positive + true negative]/[true positive + false positive+ true negative + false negative]) were hired to analyze the expression of Flot-2 protein in NPC. The results in Table 3 showed that positive expression of Flot-2 protein was one predictive factor for lymph node metastasis of NPC with good sensitivity (93%). The positive predictive value and negative predictive value of Flot-2 for lymph node metastasis of NPC was 78.2% and 53.3%, respectively. The agreement rate of Flot-2 for the assessment of lymph node metastasis of NPC was more than 60%. Furthermore, there was statistically significant difference between the positive expression of Flot-2 protein in the NPC with lymph node metastasis and those without lymph node metastasis (*P* = 0.008).

**Table 3:** Summary of statistical analysis of Flot-2 immunostaining for the detection of NPC patients with lymph node metastasis.

Sensitivity(n[%])	93/100(93)
Specificity(n[%])	8/34(23.5)
PPV(n[%])	93/119(78.2)
NPV(n[%])	8/15(53.3)
Agreement rate(n[%])	101/134(75.4)
*P value* *	0.008

*Calculated using chi-square test.

PPV: positive predictive value; NPV: negative predictive value.

## Discussion

Flotillins reside at the cytoplasmic face of the plasma membrane and at the membranes of intracellular compartments [12]. Evidence is accumulating that Flot-2 is involved in various cellular processes such as trafficking and targeted delivery of membrane and membrane protein to very specific site, T cell and B cell activation, regulation of several signaling pathways associated with cell growth and malignant transformation, keeping structure and junction of epidermal cells and formation of filopodia. Stable transfection of Flot-2 fusion gene to COS cells induced filopodia formation and changed epithelial cell to a neuronal appearance [5]. And over-expression of Flot-2 in SB2 melanoma cells facilitated the transformation of their phenotype from non-tumorigenic, non-metastatic to highly tumorigenic and metastatic in the nude mouse xenograft model [7]. Signaling cascades triggered by the lipid-modified morphogens of the Wnt family are involved in virtually all aspects of development throughout the animal kingdom. The lipid raft-associated Flotillin proteins could influence Wnt secretion and spread in Drosophila [13]. In addition to their role in Wnt secretion and spread, Flotillins have been involved in variety of membrane proteins such as cholesterol transporter [14], epidermal growth factor receptor [15] and so on. In this report, our results showed there was a high positive expression of Flot-2 protein in NPC and nasopharyngeal epithelia with atypical hyperplasia, but Flot-2 was significantly low positive expression in the control nasopharyngeal epithelia tissues, Moreover, the results indicated that the patients with the advanced clinical stages had significantly higher positive percentage of Flot-2 expression than that in the early stages of NPC. These results suggested that Flot-2 might play an important role in promoting the development and progression of NPC, especially in the early stage of NPC carcinogenesis. However, only 13 cases of nasopharyngeal epithelia with atypical hyperplasia in our research were not sufficient. So, further research should be done to investigate it with much larger samples. Also, further studies are necessary to identify the role mechanism of Flot-2 in NPC.

Invasion and metastasis are the basic biological characters which relate to recurrence and also effect on NPC patients' survival [16]–[17]. Previous studies have identified many different genes that are involved in metastasis of NPC, accelerating or suppressing metastasis by a mechanism of cooperative or inhibitive interactions between genes, such as LMP1 [18], NGX6 [19]–[20], Fibulin-5 [21], IntergrinαV[22], MIF and DJ-1[23] and so on. Our previous studies also confirmed that high expression of p-eIF4E and p-Mnk1 played a critical role in promoting invasion and metastasis and related with the poor prognosis of NPC patients [24]. The formation of filopodia is considered an important factor for promoting tumor metastasis. Reduction of Flot-2 protein levels significantly reduced the tumorigenicity and metastatic capability of a human breast cancer cell line in vivo. Flot-2 deficiency leads to a striking reduction in the number of lung metastasis, so they think that Flot-2 is an important regulator of mammary tumor-derived lung metastasis [25]. In this study, our results indicated that expression of Flot-2 protein in NPC with lymph node metastasis was significant higher than those without lymph node metastasis by univariate analysis. Multivariate analysis proved that Flot-2 positive expression was an independent predicted factor for lymph node metastasis in NPC excluding clinical stages. Therefore, these results strongly support that high expression of Flot-2 protein plays a critical role in promoting invasion and metastasis of NPC.

The sensitivity evaluates the ability of Flot-2 protein in diagnosis correctly the lymph node metastasis of NPC, and the specificity evaluates its ability about judgment in NPC patients without lymph node metastasis. The agreement rate indicates the authenticity of Flot-2 protein for lymph node metastasis of NPC. The positive predictive value indicates the possibility of diagnosis of NPC with lymph node metastasis when the expression of Flot-2 protein is positive. The negative predictive value indicates the possibility of diagnosis of NPC without lymph node metastasis when the expression of Flot-2 protein is negative. In this study, Flot-2 protein was found to show a higher sensitivity (93%) and agreement rate (75.4%) for identifying lymph node metastasis in NPC. These results indicated that Flot-2 might had more important significance in evaluating validation of Flot-2 in predicting lymph node metastasis of NPC patients. So, Flot-2 could be used as a reliable molecular marker for identifying lymph node metastasis of NPC.

There are many factors that are related with NPC prognosis. Our previous research found that high expression of β-catenin and decreased expression of E-cadherin were significantly correlated with a poor prognosis of NPC patients [26]. Over-expression of Flot-2 has been observed in several solid tumors and is correlated with poor prognosis or recurrence, metastasis in human tumors [6], [8]–[9]. Our results showed that lymph node metastasis, clinical stages were significantly correlated with patients' overall survival by univariate analysis. But univariate survival (long-rank test) analysis showed that positive expression of Flot-2 had no significant correlation with overall survival rate of NPC patients, which differed from the results of published reports about different solid tumors [8]–[9]. The most important reason may be due to the unique histological features of NPC. Non-keratinizing carcinoma including is classified into two histological subtypes according to WHO NPC classification: differentiated non-keratinized carcinoma and undifferentiated carcinoma. Non-keratinizing carcinoma is endemic in certain areas (highly in Southeast Asia) with a direct relation to Epstein-Barr virus (EBV) and has a rate as high as one-third for local recurrence or metastasis, which has high sensitivity to radiotherapy [27]–[28]. Other possible explanations for the discrepant results between NPC and breast cancer and gastric cancer may include (a) different exposure to environmental factors, such as diet or EBV infection, (b) different methods of assay, (c) differences in sampling, or population specifics and so on. Therefore, we think further large scale validation is required to evaluate the clinical value of Flot-2. Obviously, further studies will be required to elucidate the precise mechanism of Flot-2 in NPC.

In summary, we have examined the expression of Flot-2 protein in NPC, nasopharyngeal epithelia with atypical hyperplasia and control nasopharyngeal epithelial tissues. By analyzing the association between expression of Flot-2 protein and clinicopathological characteristics of NPC, we first report that high expression of Flot-2 protein was the independent predicted factor for lymph node metastasis in NPC excluding clinical stages. Therefore, increased expression of Flot-2 protein could be used as a novel biomarker for lymph node metastasis of NPC.
